# Long-term access to live black soldier fly larvae (*Hermetia illucens*) stimulates activity and reduces fearfulness of broilers, without affecting health

**DOI:** 10.1038/s41598-020-74514-x

**Published:** 2020-10-15

**Authors:** Allyson F. Ipema, Eddie A. M. Bokkers, Walter J. J. Gerrits, Bas Kemp, J. Elizabeth Bolhuis

**Affiliations:** 1 Adaptation Physiology Group, Department of Animal Sciences, Wageningen University and Research, P.O. Box 338, 6700 AH Wageningen, The Netherlands; 2 Animal Production Systems Group, Department of Animal Sciences, Wageningen University and Research, P.O. Box 338, 6700 AH Wageningen, The Netherlands; 3 Animal Nutrition Group, Department of Animal Sciences, Wageningen University and Research, P.O. Box 338, 6700 AH Wageningen, The Netherlands

**Keywords:** Physiology, Zoology

## Abstract

Commercially housed broilers frequently experience limited environmental stimulation and various health issues, compromising their welfare. Providing environmental enrichment can alleviate these problems by facilitating natural behaviour and activity. We investigated the effect of providing live black soldier fly larvae (BSFL) to broilers housed at commercial densities (33 kg/m^2^) on behaviour, fearfulness, health and performance. One-day-old broilers were distributed over five treatments with eight pens/treatment: a control treatment without BSFL; two treatments where 5% of the daily nutrient intake was replaced with live BSFL, provided four or seven times a day; and two treatments where 10% of the daily dietary intake was replaced with live BSFL provided four times a day or in transparent, movable tubes with holes. In all BSFL treatments foraging behaviour, and thereby broiler activity, was increased. Prolonged access to live BSFL, either by providing larvae seven times a day or in tubes, caused the largest increase in activity while also decreasing the time spend in tonic immobility, indicating reduced fearfulness. Broiler final weight and health were not affected. Overall, long-term access to live BSFL seems most effective in improving broiler welfare by facilitating natural behaviour and reducing fearfulness, without hindering broiler performance and health.

## Introduction

Decades-long genetic selection on increased growth rate and feed efficiency in broilers has caused broiler meat to become one of the most consumed animal meats worldwide^1^. Fast growth combined with a commercial rearing environment can compromise broiler welfare and cause low levels of activity. Broilers housed in large commercial systems spend around 70% of their time sitting^2,3^, with the highest levels of sitting occurring at the end of the production period. The legs of the inactive broilers are in long-term contact with moist and ammonia-rich litter, which can result in contact dermatitis^4,5^, including foot pad dermatitis and hock burn. Furthermore, inactivity has been linked to a range of developmental leg problems such as tibial dyschondroplasia, resulting in lameness^6,7^. Reports indicate that 20–30% of broilers suffer from moderate to severe lameness^8–10^, and as lameness can be painful^11^, limit the expression of active behaviours^12^, and reduce access to feed and water^12^, it impairs broiler welfare. Welfare problems tend to be worsened under commercial conditions with high stocking densities, restricted space, and minimal environmental stimulation, as these conditions limit the broiler’s range of behavioural choices and can cause elevated levels of fearfulness^13,14^.

Providing environmental enrichment that facilitates a broader range of behaviours might increase broiler activity, resulting in improved leg health and broiler welfare. However, in previous studies enrichment materials such as straw bales, perches or strings, had varying effects on broiler activity and welfare (for review see refs^15,16^). In a recent study, foraging behaviour and activity of broilers were successfully increased by means of lasers which moved around the pen four times a day in 4-min periods^17^. While this type of enrichment increases broiler activity, facilitating foraging behaviour without including a rewarding aspect (e.g. consumption of a feed item) could cause frustration. Providing a preferred feed item as enrichment could increase foraging behaviour without causing frustration. Also, foraging for and consuming a preferred feed item can be rewarding and positively influence dopaminergic systems in the broiler’s brain^18^, and through this promote a more positive affective state^19^. Laying hens with outdoor access showed both a more positive affective state and lower levels of fearfulness than layers kept indoors, and if a similar link can be found in broilers, providing preferred feed items could reduce broiler fearfulness through improving the affective state^20^.

Feed items that could be used as effective enrichment are insects, which broilers are highly motivated to interact with and consume^21^. Insects are part of the natural diet of chickens, and several insect meals have been included in broiler feed without negatively affecting broiler health and/or performance^22–24^. Providing a small amount of mealworms increased broiler activity for several minutes^25^. In our previous study, replacing 5 or 10% of the dietary dry matter intake of broilers with live black soldier fly larvae (BSFL) and scattering them across the pen twice or four times a day promoted active behaviours. Furthermore, providing live BSFL four times a day reduced the occurrence of lameness and hock burn without affecting performance^26^. While BSFL provisioning successfully promoted activity, the broilers in this study were kept at low stocking densities (4.5 broiler/m^2^)^26^. Stocking density can affect broiler behaviour and welfare^27^. Therefore, broilers might respond differently to insect provisioning when housed at commercial densities. Furthermore, several authors have suggested prolonging the period during which broilers have access to insects, as this can amplify the increase in broiler activity, as well as the resulting welfare benefits^25^. Prolonging the access duration could have the additional benefit of allowing broilers to habituate more to the presence of novel items that do not require a fear response, potentially reducing broiler fearfulness. Currently, it is unclear whether prolonging access to live BSFL expands the welfare benefits for broilers, and which methods for prolonging access to insects are most effective in improving broiler welfare.

The aim of this study was to determine the effect of prolonged access to live BSFL on the behaviour, fearfulness, health and performance of broilers kept under commercial densities (33 kg/m^2^), while maintaining a similar nutrient intake between treatments. We hypothesized that prolonged access to live BSFL would cause the largest activity increase, fearfulness reduction, and health improvement compared to controls.

## Methods

The Animal Care and Use committee of Wageningen University & Research approved the experimental protocol under project licence number 2018.W-0036, and the protocols were in accordance with the Dutch law on animal experimentation, which complies with European Directive 2010/63/EU. The experiment was executed at the animal experiment facilities of Wageningen University & Research (Wageningen, The Netherlands).

### Animals and management

At arrival, 880 1-day old male Ross 308 broilers received a neck tag after which they were randomly distributed over forty pens, resulting in 22 broilers/pen. Ten randomly selected focal broilers per pen were marked with a coloured dot (stock marker) for individual identification. Each pen was 1 × 2 m in size and contained two feeders, five drinking nipples with cups, one 1 m long perch (rectangular bar of 2 × 2 cm, 10 cm high) and a 5 cm deep layer of wood shavings. Feed and water were provided ad libitum. The lighting schedule (20 lx at chick level) was 1D:23L on day 1–3, after which it was gradually decreased to 6D:18L and kept constant throughout the remainder of the experiment. Temperature and humidity were based on the Aviagen Ross broiler handbook. At hatch, all chicks received an IB vaccination (spray) and on day 16 they received NCD vaccination (spray).

### Experimental design

The study included one control treatment without BSFL provisioning and four treatments with live BSFL provisioning. All treatments were designed to maintain identical nutrient composition of the total feed plus live BSFL offered, as outlined below. The two most effective treatments from our previous study^26^, i.e. replacing 5 or 10% of the dietary dry matter (DM) intake with live BSFL scattered throughout the pen in four equal portions a day, were included in the current study to determine their effect under commercial stocking densities. Two additional treatments were included; one in which 5% of the dietary DM intake was replaced with live BSFL, scattered throughout the pen in seven equal portions a day, and one in which 10% of the dietary intake was replaced with live BSFL, provided in transparent tubes with holes. Scattering a small amount seven times a day reduces portion size, making the larvae harder to locate, and providing larvae in tubes requires broilers to actively manipulate the tubes to acquire the live larvae. These treatments were chosen with the aim of prolonging access to the live larvae. Tubes were filled every morning with the total daily amount of live BSFL, and broilers had continuous access to the tubes. Based on a pilot study, tube size and the number of holes in the tubes were chosen as they resulted in limited larvae release solely by larvae activity, meaning that broilers had to actively get the live larvae out of the tubes. Tube dimensions and holes were adjusted throughout the experimental period to complement the increasing broiler size and the increasing BSFL amount (Supplementary Table S1). In the remainder of the text, all BSFL treatments will be referred to by the BSFL amount (5 or 10%, called A5 and A10) and BSFL delivery method (scattered four or seven times a day, called S4 and S7; and in tubes, called TB, Supplementary Table S2). Each treatment was applied to eight pens, which were equally distributed over eight blocks, with four blocks per experimental room (Supplementary Table S3). A commercial BSFL producer supplied live 14-day-old BSFL each week, which were stored at 12 °C near the experimental rooms until use.

### Diet composition

All diets met or exceeded the broiler’s nutrient requirements^28^. From day 1–7, broilers received a starter feed (12.46 ME/kg DM, 22% crude protein) that was not adjusted for larval intake as the digestible nutrient intake from BSFL was expected to be low for young broilers. From day 8–42, broilers received a grower feed which was adjusted to the estimated larvae intake to ensure a similar nutrient and energy intake between treatments. The grower feed was designed as follows. First, three samples of BSFL were analysed for dry matter, crude protein, crude fat, calcium and phosphorus content. Based on this analysis, a feed mix was designed mimicking the nutritional composition of BSFL. A basal grower feed containing 10% of this mix was provided to control broilers. The grower feeds for the A5 and A10 broilers were designed by omitting half or all this mix from the basal feed, respectively (nutrient composition in Supplementary Table S4). Results from the BSFL analysis were also used to determine the daily portions of live larvae.

### Measurements

#### Behavioural observations

The behaviour and posture of focal broilers were observed in 8-min intervals by instantaneous scan sampling 1 day in week 1–5 (day 5, 12, 19, 26, and 33) according to the ethogram in Table 1. Each day had seven 64-min observation periods, and each period consisted of one scan before and seven scans after the time of larval provisioning (of the A5-S7 group, as not all treatments received larvae in each observation period). Four observers simultaneously observed 10 pens each, and every observation period the observers switched between pens. The observers were trained, and inter-observer reliability was deemed to be sufficient (Fleiss kappa > 0.8), before observations took place.

**Table 1:** Ethogram of behavioural observations.

Item	Description
**Behaviour**	
Eating	Having head above or in the feeder and/or pecking at feed in the feeder or on the floor
Drinking	Drinking from nipple or cup beneath nipple
Locomotion	Walking (locomoting in upright position with a normal speed or quick steps) or shuffling (half standing/half sitting and moving a few steps before sitting down), without performing any other behaviour
Defecation	Excreting faeces
Standing idle	Standing on the ground without performing any other behaviour
Perching	Perching without performing any other behaviour
Resting	Sitting with hocks resting on ground without performing any other behaviour, possibly with head on the ground or under wing
Scratching	Scraping of the litter with the claws
Ground pecking	Performing pecking movements directed at the ground
Food running	Running with food in beak while pen mates follow and attempt to grab the food item
Dust bathing	Performed with fluffed feathers while lying, head rubbed on floor, wings opened, scratching at ground, distributing substrate over body
Stretching	Stretching of wing and/or leg
Preening	Grooming of own feathers with beak
Wing flapping	Bilateral up-and-down wing flapping
Agonistic	Jumping at pen mate, threatening pen mate, pecking movements directed at head of pen mate
Pecking pen mate	Pecking movements directed at the body or beak of pen mate
Interaction tube	Pecking movement directed at tube or moving tube
Other	Any behaviour not mentioned above
**Posture**	
Standing	On floor: hocks not in contact with the litter. On perch: knees not bent
Sitting	On floor: hocks in contact with the litter. On perch: knees bent

#### Tonic immobility test

On day 15 and 16 between 08:30 and 12:30, four focal birds per pen were subjected to a tonic immobility (TI) test, a well-validated test used to assess chicken fearfulness^29^. Broilers were tested in a randomized order balanced for treatment, in a separate room close to the experimental rooms. Each broiler was placed on its back on a table with its head hanging over the edge of the table. To induce TI, the experimenter restrained the bird for 15 s by placing one hand on its sternum and one on its head covering the eyes. After induction, the experimenter slowly stepped back and the latency to vocalize, move their head and stand up were recorded. If the bird stood up within 10 s, the induction attempt was immediately repeated. If the bird stood up within 10 s on three attempts, this was noted, and the test was terminated. If a bird was still in TI after ten minutes, it received the maximum score of 600 s.

#### Health measurements

On day 40, all 10 focal broilers per pen were visually scored on their degree of hock burn, foot pad dermatitis, lameness, cleanliness and thigh scratches. On day 42, six focal broilers per pen were sacrificed and analysed post-mortem. Due to mechanical malfunction, half of the birds per pen were euthanised by electrocution, and half were euthanised by lethal injection with Euthasol, balanced per treatment. Broiler body and heart weight were measured, and the degree of white striping and wooden breast of the pectoral muscles, and the presence of abdominal fluid were scored. The legs of the sacrificed broilers were removed and stored at − 20 °C for several days. After thawing, the tibias were removed, and tibia proximal length and lateral cortex width were measured with a digital calliper. Fluctuating asymmetry was calculated by dividing the absolute length difference between the left and right tibia by the average length of the left and right tibia. A veterinarian scored tibial dyschondroplasia on the left tibia by making a diagonal cut in the femoral proximal head and scoring lesions (for full descriptions of scores, see Supplementary Table S5)^30–32^. The breaking strength (maximum load to break the tibia in Newton) of the right tibia was determined using an Instron Electromechanical Universal Testing Machine (Instron, Norwood, MA, USA).

#### Litter quality

On day 41, the level of litter friability and wetness were scored visually in each pen based on the protocol described by van Harn et al.^33^. Additionally, litter samples from under the drinking line, next to the feeder and in the centre of each pen were taken and mixed, and the moisture percentage was determined by weighing the litter sample before and after drying at 70 °C for 24 h.

#### Performance

All broilers were weighed before placement and on day 7, 14, 21, 28, 35 and 41, and based on this the average daily gain (ADG) per broiler was calculated. Feed consumption on pen level was determined over the entire growing period (day 8–42). Regular observations indicated that most of the live larvae were consumed each day and based on this the percentage of the total DM intake consisting of BSFL was determined per pen. Morbidity and mortality were recorded daily, and the amount of live larvae provided was adjusted if chick mortality occurred.

### Statistical analysis

#### Data processing

For the grower period the average daily dietary DM (with and without BSFL) in g/day/chick was determined and based on this the average daily energy intake per chick was calculated. Observed behaviours and postures were grouped per chick per day and expressed as the percentage of observations in which the behaviour occurred. All behaviours occurring in more than 0.5% of the observations (eating, drinking, locomotion, standing idle, perching, resting, scratching, ground pecking, stretching, and preening) were analysed independently. Additionally, the grouped items “foraging behaviour” (ground pecking, scratching, food running and interaction tube), “comfort behaviour” (preening and dust bathing) and “activity” (all behaviours except resting and sitting while perching) were analysed. Foot pad dermatitis, wooden breast and abdominal fluid were uncommon (< 2%) and the effect of treatment on these parameters was therefore not analysed. Per chick only the leg with the highest hock burn score was included in the analysis. Hock burn scores of 3 and 4 and gait scores of 4 and 5 were rare (< 1%) and therefore these scores were grouped with a score of 2 for hock burn and a score of 3 for gait score. All broilers had a white striping score of 1 or 2, therefore this was analysed as a binary variable.

#### Data analysis

Data were analysed using SAS 9.4 (SAS Institute Inc., Cary, NC, USA). All mixed models included block and pen (nested in block and treatment) as random effects. Behaviours were analysed with a generalized linear mixed model (GLIMMIX in SAS) with a binomial distribution, logit link function and additional multiplicative over-dispersion parameter. Here, the fixed effects were treatment, week and the treatment by week interaction. For the behaviour “interaction tube” the model included only week as fixed effect as only A10-TB birds could perform this behaviour. A random effect of week with chick as subject (nested within pen, block and treatment) was added, with a heterogeneous first-order autoregressive covariance structure. Tonic immobility latencies, health scores and performance parameters were analysed with treatment as fixed effect and density as covariate. Ordinal scores were analysed in a GLIMMIX with either a binary or multinomial distribution, and continuous scores were analysed in a MIXED procedure. Tonic immobility latencies and fluctuating asymmetry values were square root transformed for normalization. Visual litter friability and wetness scores were analysed with a Kruskal–Wallis test due to the occurrence of empty subclasses. The proportion of moisture was analysed with a GLIMMIX with binomial distribution and additional over-dispersion parameter. Significant treatment effects were further analysed using differences in least square means with a Tukey’s HSD correction or, for visual litter quality scores, a Dwass–Steel–Critchlow–Fligner multiple comparisons test. Data are presented as means ± SEM based on pen averages. *P* values below 0.05 were considered statistically significant.

## Results

### Behavioural observations

**Figure 1:**
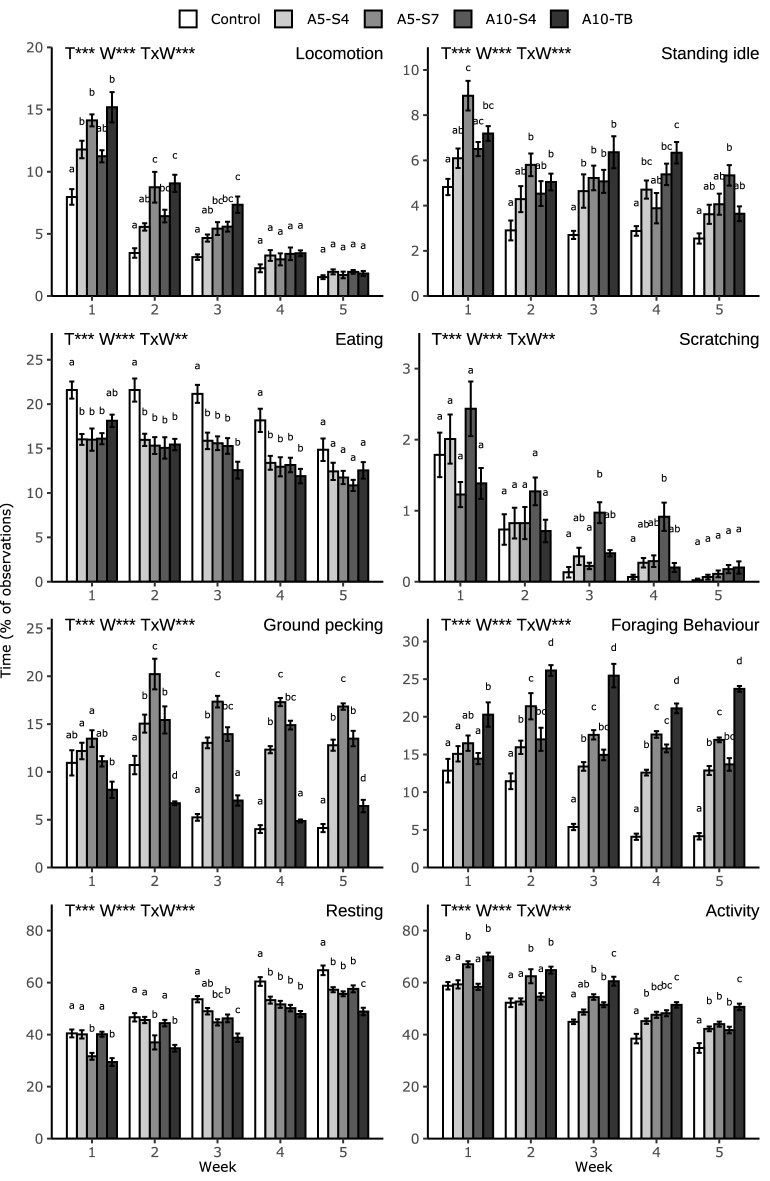
Behavioural activities (% of observations) of broilers receiving no larvae (Control), or provided with live larvae in different amounts (5 or 10% of the total dietary DM replaced with larvae, A5 and A10 respectively) and provisioning methods (scattered four or seven times a day, S4 and S7 respectively, or in tubes, called TB) scored in week 1–5. Foraging behaviour encompasses ground pecking, scratching, food running and interaction with tube. Activity encompasses all behaviours except resting and sitting while perching. Data presented as means ± SEM. Effects of Treatment (T), Week (W) and their interaction (T × W) are indicated as **p < 0.01 or ***p < 0.001. Different letters within 1 week indicate significant (*p* < 0.05, Tukey’s HSD correction) differences between treatments.

Treatment did not influence the percentage of time spent perching (1.21 ± 0.06%, *p* > 0.05) and stretching (0.89 ± 0.03%, *p* > 0.05). The behaviours locomotion, standing idle, eating, scratching, ground pecking, foraging behaviour, resting and total activity were influenced by treatment, week and the treatment by week interaction (*p* < 0.01 for all, Fig. 1). Treatment and week, but not the treatment by week interaction, affected drinking, preening and comfort behaviour, and week affected the time spent on interacting with the tubes (*p* < 0.001). Pairwise significant (*p* < 0.05) differences are discussed below.

#### Locomotion

In week 1, broilers in treatments A10-TB, A5-S7, and A5-S4 walked more than control broilers. During week 2 and 3 A10-TB, A5-S7 and A10-S4 broilers walked more than controls, and in these two weeks A10-TB broilers also walked more than A5-S4 broilers. Over time, the stimulation of locomotion by live BSFL provisioning decreased, until no treatment effects were observed in week 4 and 5.

#### Standing idle

During one or several weeks broilers in all BSFL treatments spent more time standing idle than control broilers (A5-S4: week 3 & 4, A10-S4: week 3–5, A5-S7: week 1–3, A10-TB: week 1–4). In week 1, A5-S7 broilers spent more time standing idle than A5-S4 broilers, and in week 4 A10-TB broilers stood idle more than A5-S7 broilers. The time spent standing idle declined in week 5 for A5-S4 broilers, while for the other treatments this decline occurred first in week 2. After the initial decline the time spent standing idle remained relatively constant for broilers in the control, A5-S7 and A10-S4 treatments. In contrast, for A10-TB broilers the initial decline was followed by an incline in time spent standing idle in week 3, and this was again followed by a decline in time spent standing idle in week 5.

#### Eating and drinking

In the first four weeks, control broilers spent more time eating feed than those in all BSFL treatments, except for A10-TB broilers in week 1. In week 5, treatment did not affect time spent eating. The time spent eating feed of all broilers decreased over time, independent of the live BSFL amount. Controls spent more time drinking than broilers in the BSFL treatments (C = 5.5 ± 0.3%, A5-S4 = 4.0 ± 0.2%, A5-S7 = 4.2 ± 0.2%, A10-S4 = 3.8 ± 0.2%, A10-TB = 3.8 ± 0.2%, *p* < 0.001) and for all treatments the time spent drinking decreased steadily during week 1 until and including week 3, after which it increased again in week 5.

#### Foraging behaviour

Time spent interacting with the tubes (which could only be performed by A10-TB broilers) was lower in week 1 compared to all other weeks, and in week 4 compared to week 2 and 3. The A10-S4 broilers spent more time scratching than A5-S7 broilers in week 3 and than controls in week 3 and 4, and all treatments showed variations in time spent scratching over time. In week 2–5, A5-S4, A5-S7 and A10-S4 broilers spent more time on ground pecking than controls and A10-TB broilers. Additionally, A5-S7 broilers spent more time on ground pecking than A5-S4 broilers in week 2–5 and A10-S4 broilers in week 2 and 5. In week 2, controls also spent more time on ground pecking than A10-TB broilers, and in week 5 this was reversed. Overall, all methods of live BSFL provisioning persistently increased the time spent on foraging behaviour. The effects on foraging behaviour were similar as for ground pecking, except that A10-TB broilers foraged more than controls, A5-S4 and A10-S4 broilers in all weeks, and they foraged more than A5-S7 broilers in week 2–5. The control broiler’s time spent ground pecking and total foraging behaviours decreased after two weeks after which it remained constant. In contrast, A5-S4, A5-S7 and A10-S4 broilers showed a relatively constant high level of ground pecking and foraging behaviour throughout all weeks. While A10-TB broilers spent a consistently low amount of time on ground pecking, their level of total foraging behaviour started high and increased further in week 2, 3 and 5 compared to week 1.

#### Resting and activity

The patterns for resting and activity were almost complementary as activity consists of all behaviours except resting and sitting while perching, therefore only activity is discussed. During several weeks control broilers were less active than broilers in the BSFL treatments (A5-S4: week 4 & 5, A10-S4: week 3–5, A5-S7 & A10-TB: week 1–5). In addition, A10-TB broilers were more active than A5-S4 broilers during all weeks, and A5-S7 broilers were more active than A5-S4 broilers in week 1 and 2. Birds from all treatments showed a decline in activity over time, and this decline started in week 2 for control, A5-S4 and A10-TB broilers, and in week 3 (compared to week 1) for A5-S7 and A10-S4 broilers.

#### Comfort behaviour

A10-TB broilers spent less time on preening (C = 4.0 ± 0.2%, A5-S4 = 4.2 ± 0.2%, A5-S7 = 3.9 ± 0.2%, A10-S4 = 4.1 ± 0.2%, A10-TB = 3.2 ± 0.2%, *p* < 0.001) and total comfort behaviour (C = 4.3 ± 0.3%, A5-S4 = 4.5 ± 0.2%, A5-S7 = 4.2 ± 0.2%, A10-S4 = 4.4 ± 0.2%, A10-TB = 3.4 ± 0.2%, *p* < 0.001) than broilers in all other treatments. The time spent on comfort behaviours decreased in week 2 compared to week 1, and in week 3 compared to week 2, after which it remained constant.

### Posture

**Figure 2:**
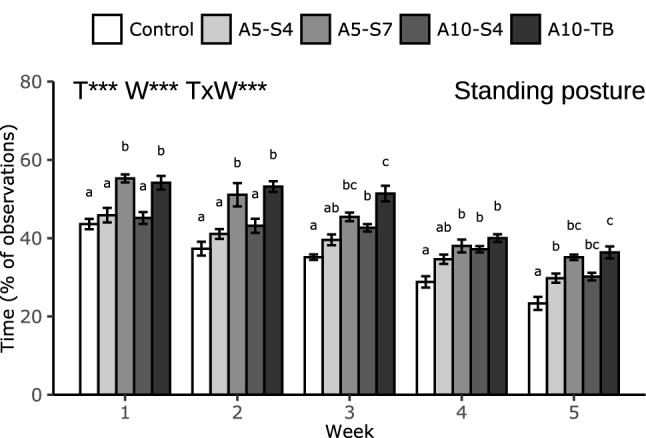
Time spent in standing posture (% of observations) of broilers receiving no larvae (Control), or provided with live larvae in different amounts (5 or 10% of the total dietary DM replaced with larvae, A5 and A10 respectively) and provisioning methods (scattered four or seven times a day, S4 and S7 respectively, or in tubes, called TB) scored in week 1–5. Data are presented as means ± SEM. Effects of Treatment (T), Week (W) and their interaction (T × W) are indicated as ***p < 0.001. Different letters within one week indicate significant (*p* < 0.05, Tukey’s HSD correction) differences between treatments.

Treatment, week and the treatment by week interaction affected posture (*p* < 0.001 for all, Fig. 2). During several weeks, controls stood less than broilers in the BSFL treatments (A5-S4: week 5, A10-S4: week 3–5, A5-S7 & A10-TB: week 1–5). A10-TB broilers spent more time standing than A5-S4 broilers during all weeks except week 4, and A5-S7 spent more time standing than A5-S4 broilers in week 1 and 2. Birds in all treatments showed a decline in time spent standing over time. For the controls this decline started in week 2 (compared to week 1), for A5 broilers the decline started in week 3, and for A10 broilers the decline started in week 4.

### Tonic immobility

Treatment influenced the time spent in tonic immobility, where controls stayed in tonic immobility longer than A5-S7 and A10-TB broilers, with the A5-S4 and A10-S4 in between. Treatment did not affect latency to vocalize and latency to first head movement (Table 2).

**Table 2:** Tonic immobility (TI) responses of broilers receiving no larvae (Control), or provided with live larvae in different amounts (5 or 10% of the total dietary DM replaced with larvae, A5 and A10 respectively) and provisioning methods (scattered four or seven times a day, S4 and S7 respectively, or in tubes, called TB). Broilers that were not induced in tonic immobility after three attempts, and broilers that did not vocalize or move their heads were excluded from the respective analysis. Data are presented as means. Different letters indicate significant (*p* < 0.05, Tukey’s HSD correction) differences between treatments. *p < 0.05.

Measure	Control	A5-S4	A5-S7	A10-S4	A10-TB	SEM	F-statistic and df	P
Time in TI (s)	183.8*a*	119.8*ab*	95.6*b*	168.2*ab*	103.0*b*	12.0	F(4,35) = 3.87	0.010*
Latency to vocalize (s)	33.6	25.0	36.2	47.7	14.9	5.6	F(4,33) = 1.04	0.400
Latency to move head (s)	54.6	24.9	17.4	20.4	21.0	4.2	F(4,28) = 1.15	0.356

### Health

Neither hock burns, lameness, cleanliness, thigh scratches and white striping (Table 3), nor the broilers’ heart weight, tibia length, tibia fluctuating asymmetry and tibia breaking strength (Table 4) were influenced by treatment. Treatment did influence broiler tibia width (*p* = 0.024), where the tibia of controls were wider than those of A10-S4 broilers.

**Table 3:** Frequencies of health scores of broilers receiving no larvae (Control), or provided with live larvae in different amounts (5 or 10% of the total dietary DM replaced with larvae, A5 and A10 respectively) and provisioning methods (scattered four or seven times a day, S4 and S7 respectively, or in tubes, called TB). Health parameters were analysed with linear mixed models. Foot pad dermatitis, wooden breast and abdominal fluid levels were not analysed due to low variance.

Measure	Score	Control	A5-S4	A5-S7	A10-S4	A10-TB	F-statistic and df	P
Gait	0	8	5	5	8	5	F(4,34) = 0.48	0.750
	1	22	24	26	27	33		
	2	42	42	44	40	35		
	3	8	8	5	5	5		
Hock burn	0	35	49	38	40	41	F(4,34) = 0.85	0.506
	1	38	23	33	27	33		
	> 1	7	8	9	13	6		
Foot pad dermatitis	0	79	80	78	75	80	–	–
	1	1	0	2	5	0		
Cleanliness	1	6	3	3	9	2	F(4,35) = 0.21	0.933
	2	35	47	49	29	40		
	3	39	30	28	42	37		
Thigh scratches	Absent	61	66	68	63	57	F(4,35) = 1.13	0.357
	Present	19	14	12	17	22		
Wooden breast	Absent	43	45	48	48	48	–	–
	Mild	4	3	0	0	0		
	Severe	1	0	0	0	0		
White striping	Mild	22	24	32	26	27	F(4,35) = 1.08	0.382
	Severe	26	24	16	22	21		
Abdominal fluid	Absent	47	48	48	47	48	–	–
	Present	1	0	0	1	0		
Tibial dyschondroplasia	Absent	35	27	31	27	29	F(4,35) = 0.94	0.453
	Present	13	21	17	21	19

**Table 4:** Heart and tibia measurements of broilers receiving no larvae (Control), or provided with live larvae in different amounts (5 or 10% of the total dietary DM replaced with larvae, A5 and A10 respectively) and provisioning methods (scattered four or seven times a day, S4 and S7 respectively, or in tubes, called TB). All parameters analysed by linear mixed models (fluctuating asymmetry (FA) was first square root transformed). Within each row different letters indicate significant (*p* < 0.05, Tukey’s HSD correction) differences. *p < 0.05.

Measure		Control	A5-S4	A5-S7	A10-S4	A10-TB	SEM	F-statistic and df	P
Heart weight (g)		11.6	11.6	11.9	11.6	11.8	0.1	F(4,35) = 0.19	0.943
Tibia measures	Length (mm)	102.2	101.8	102.3	101.3	101.3	0.2	F(4,35) = 0.93	0.460
	Width (mm)	8.7*a*	8.6*ab*	8.5*ab*	8.2*b*	8.4*ab*	0.05	F(4,35) = 3.23	0.024*
	Length FA (mm)	0.6	0.5	0.5	0.5	0.5	0.03	F(4,35) = 0.31	0.869
	Width FA (mm)	0.3	0.3	0.2	0.2	0.3	0.02	F(4,35) = 0.11	0.980
	Breaking strength (N)	445.2	471.2	449.9	448.8	463.4	5.1	F(4,35) = 0.87	0.492

### Litter quality

Treatment influenced the friability of the litter (*p* = 0.008). The A10-S4 pens had less friable litter than control and A10-TB pens. The visual wetness score and the proportion of moisture in the litter were not affected by treatment (Table 5).

**Table 5:** Litter quality scores of pens with broilers receiving no larvae (Control), or provided with live larvae in different amounts (5 or 10% of the total dietary DM replaced with larvae, A5 and A10 respectively) and provisioning methods (scattered four or seven times a day, S4 and S7 respectively, or in tubes, called TB). Visual friability and wetness scores were analysed with a Kruskal–Wallis test and a post-hoc DSCF test and moisture percentage was analysed with a generalized linear mixed model. ^1^sum of scores calculated by a Kruskal–Wallis test. **p < 0.01.

Measure	Score	Control	A5-S4	A5-S7	A10-S4	A10-TB	SEM	Test statistic and df	P
Visual friability score (frequency of scores)	2	0	1	3	5	0	-	H(4) = 13.72	0.008**
	3	4	3	4	3	4			
	4	3	4	0	0	4			
	5	1	0	1	0	0			
	Sum of scores^1^	2130*a*	193*ab*	129*ab*	81*b*	206*a*			
Wetness score (frequency of scores)	2	0	0	0	1	1	–	H(4) = 3.88	0.423
	3	6	4	3	5	4			
	4	2	4	5	2	3			
	Sum of scores^1^	146	184	203	134	153			
Moisture (%)	Mean	56.3	57.8	58.4	60.4	55.9	0.7	F(4,35) = 1.25	0.320

### Performance

Over the course of the experiment, several broilers were removed due to health problems: 3 broilers from the control, A5-S4, A5-S7 and A10-S4 treatments, and 4 broilers from the A10-TB treatment. In the third week, broilers in both A10 treatments had a lower ADG than controls, and in the fourth week broilers in the A10-S4 and A4-S7 treatments had a lower ADG than controls. In week 1, 2, 5 and 6, broilers showed no differences in ADG, and their final weight was also not affected by treatment. Considering feed intake, the percentage of the total dry matter (DM) intake consisting of BSFL was 6.3 ± 0.1% for A5-S4 broilers, 6.4 ± 0.1% for A5-S7 broilers, 13.8 ± 0.2% for A10-S4 broilers and 13.8 ± 0.1% for A10-TB broilers. The daily dietary DM intake excluding BSFL dropped proportionally with the amount of live BSFL offered, where it was higher in control broilers than in all other broilers and in A5 broilers than in A10 broilers. The average daily DM and energy intake including BSFL did not differ between treatments (Table 6).

**Table 6:** Performance parameters of broilers receiving no larvae (Control), or provided with live larvae in different amounts (5 or 10% of the total dietary DM replaced with larvae, A5 and A10 respectively) and provisioning methods (scattered four or seven times a day, S4 and S7 respectively, or in tubes, called TB). All parameters were analysed with linear mixed models. Within each row different letters indicate significant (*p* < 0.05, Tukey’s HSD correction) differences. **p < 0.01, ***p < 0.001.

Measure		Control	A5-S4	A5-S7	A10-S4	A10-TB	SEM	F-statistic and df	P
Average daily gain (g/d)	W1	12.1	12.4	11.7	12.8	12.2	0.15	F(4,35) = 1.50	0.225
	W2	41.0	41.0	38.4	39.7	38.6	0.44	F(4,34) = 1.68	0.176
	W3	63.5*a*	61.2*ab*	60.1*ab*	58.4*b*	57.6*b*	0.50	F(4,34) = 5.89	0.001**
	W4	88.9*a*	87.0*ab*	82.8*b*	82.1*b*	83.8*ab*	0.75	F(4,35) = 4.06	0.009**
	W5	104.2	107.5	103.3	102.8	103.3	0.92	F(4,34) = 1.85	0.143
	W6	125.5	128.5	125.6	125.7	130.5	1.25	F(4,34) = 0.59	0.673
Final weight (g)		2,963.5	2,970.6	2,874.4	2,873.9	2,918.0	17.0	F(4,34) = 1.58	0.201
Feed intake (g/d)	Excl. BSFL	96.5*a*	92.2*b*	91.2*b*	85.2*c*	84.8*c*	0.83	F(4,34) = 23.36	< 0.001***
	Incl. BSFL	96.5	98.1	97.0	96.9	96.6	0.44	F(4,34) = 0.43	0.789
Energy intake incl. BSFL (Mj/d)		1.19	1.21	1.21	1.21	1.21	0.005	F(4,34) = 0.67	0.616

## Discussion

The current study showed that providing broilers with 5 or 10% of their dietary dry matter (DM) intake as live BSFL four times a day increased activity during the second half of the production period, mainly by facilitating foraging behaviour. Moreover, increasing the frequency of live BSFL access by providing larvae seven times a day or prolonging live BSFL access by offering them in tubes with holes increased broiler activity throughout the entire production period, while it reduced broiler fearfulness. A temporarily reduced growth rate was observed in A5-S7, A10-S4 and A10-TB broilers, but this did not influence the final weight of broilers. Broiler health was not affected by live BSFL provisioning.

The observed increase in foraging behaviour, activity, and time spent standing in broilers receiving live larvae compared to controls was in line with previous studies showing short-term effects of scattering mealworms^25^ and long-term effects of scattering BSFL^26^. Our previous study already indicated positive effects of live BSFL provisioning on activity of broilers housed at low densities, and the current study demonstrates that under commercial densities this effect is still present. As foraging is considered a behavioural need^34^, being rewarding^35^ for chickens, the welfare of these broilers is improved. Previous studies in which maize roughage or sand and strings were provided to broilers also found an increase in foraging behaviour^36,37^, however this increase was numerically lower and occurred for a shorter time compared to the current study. The difference in foraging behaviour between studies could be attributed to the high motivation of broilers to gain access to high-value feed items such as insects^21,25^ compared to items with a low nutritional value, making them relatively more willing to work for access to larvae. This is supported by the observation that overall locomotion was increased by live larvae provisioning but not by providing maize roughage or sand and strings^36,37^. In addition, the provisioning methods used in our study i.e. scattering live larvae throughout the pen or providing live larvae in tubes, required more foraging and/or locomotion to obtain the resource compared to providing stationary, easily accessible items, independent of the type of resource.

Overall activity of A5-S4 and A10-S4 broilers was only increased during the second half of the production period. This is in line with our previous study in which these treatments were applied to broilers kept at lower (14 versus 33 kg/m^2^) densities^26^. Furthermore, in the current experiment these broilers had almost identical behavioural time budgets, suggesting that frequency is more important than amount when it comes to promoting broiler activity. In line with this, increasing the frequency to seven times a day or prolonging access to live BSFL by providing them in tubes not only caused a higher increase in foraging behaviour and activity, but also increased activity during more weeks. This is in accordance with previous studies that suggested^25^ or showed^26^ that prolonging access to insects would increase broiler activity even more. Prolonged access can increase activity in multiple ways. In our study, a higher frequency of live larvae provisioning meant that broilers received smaller portions and were stimulated to be active multiple times a day. Scattering fewer live larvae throughout the pen makes larvae harder to locate, which promotes foraging behaviour. Furthermore, BSFL have a high water content of approximately 65%, and consuming high amounts of water in a short amount of time could increase satiety in broilers^38^, reducing their motivation to forage. When providing smaller portions this effect is less likely to occur. Also, at a higher frequency of live larvae provisioning the disturbance caused by providing the enrichment could also have increased broiler activity. Though, this is unlikely as broiler activity was not increased by providing wheat or wood shavings^25^, showing that disturbance alone does not promote broiler activity. For live larvae provisioning in tubes, obtaining BSFL requires substantial effort of manipulating the tube, therefore more active behaviour is required to consume the same amount of larvae. As frequent or prolonged access appears key in increasing broiler activity, further research should focus on alternative, cost- and labour-efficient methods to achieve this. For example, a recent study tested a device that allowed for gradual live BSFL provisioning to layer hens^39^, and such a device could also be applied in the broiler industry. Also, further research is required to determine the effect of BSFL amount on the success of these methods. The observation that performance was not hindered by live larvae provisioning up to 10% despite the increased activity and decreased pellet intake indicates that BSFL are a good source of nutrients and could even be provided in higher amounts, potentially increasing the welfare benefits.

The investigated live larvae provisioning methods had differential effects on individual behaviours. Scattering live BSFL resulted mainly in increases in ground pecking. Providing live BSFL in tubes resulted in a large amount of time spent interacting with these tubes, and we observed that the interaction consisted mainly of pecking and scratching at the tubes (Supplementary Video S1). Pecking at the ground or at objects is a natural behaviour of chickens^40^, therefore performing this behaviour could satisfy a motivational need of broilers. Interacting with a plastic tube might be less natural for broilers, though broilers were clearly motivated to interact with the tubes as reflected by the large amount of time they spent performing foraging-related behaviour directed towards the tubes. Therefore, broilers seem to adjust their behaviours to the environment, and both methods of provisioning can satisfy their behavioural needs. Despite differences in the target of the foraging behaviour, both more frequent and prolonged access treatments caused a similar higher increase in time spent in standing posture occurring during more weeks compared to the lower frequency treatments and controls. This is in line with a study showing that increasing standing activity from a young age onwards caused long-term increases in activity^41^. Finally, comfort behaviour occurred significantly less in broilers receiving live BSFL in tubes compared to controls. Comfort behaviour is often considered as a sign of good welfare^42^, therefore this could indicate that these birds experience diminished welfare. However, as these broilers spent between 20–25% of their time on foraging behaviour, which is considerably more than broilers kept under regular commercial conditions^2, 3^, foraging behaviour might occur at the expense of comfort behaviour. It is therefore unlikely that the reduced time spent on comfort behaviour is an indicator of diminished welfare in broilers receiving live BSFL, but rather a consequence of the increased foraging possibilities.

Providing 5% of the diet as live BSFL given seven times a day or providing 10% of the diet as live BSFL in tubes both reduced the time broilers spent in tonic immobility compared to controls, which indicates reduced fearfulness^29^. While previously tested enrichment methods such as providing mealworms once a day or adding strings and perches did not affect fearfulness^25,43^, adding perches in combination with peat dust baths did reduce fearfulness in broilers compared to barren-housed controls^2^. Prolonged access to preferred enrichment such as peat dust baths or larvae could have extended the interaction with non-threatening stimuli and adapted the birds to potentially frightening situations^44^, reducing their tonic immobility response. However, providing live BSFL four times a day did not influence broiler fearfulness, indicating that four provisioning moments are not enough to familiarize the birds to novelty. In addition, the increased interaction with live larvae could have promoted a more positive affective state in broilers through positive influences on the dopaminergic systems^18^. A more positive affective state could be linked to reduced fearfulness, as shown in layer hens^20^, though the cause and effect relationship is not completely clear.

Considering the observed health parameters, only tibia width was influenced by live BSFL provisioning, where control broilers had wider tibia than broilers receiving 10% of their diet as live BSFL four times a day, which could indicate better leg health of the controls. The A10-S4 broilers grew slower than controls during week 3 and 4 of the experiment, suggesting broiler growth rate could be linked to tibia development. In line with this, feed restriction during the second week of the production period also slowed broiler growth and reduced tibia length and width^45^. However, other studies found no relation between growth rate and tibia development^46,47^. Alternatively, the increased activity of the A10-S4 broilers compared to controls could have reduced tibia width, as Foutz et al.^48^ found that increasing broiler activity through treadmill training reduced tibia length and width^48^. However, as broilers in the other BSFL treatments also had increased activity without showing differences in tibia measures compared to controls, this cannot be the sole explanation.

The absence of a treatment effect on the other examined health parameters was unexpected, as activity was increased by live BSFL provisioning, and increased activity has positively affected walking ability and bone development in previous studies^7,41,49^. The occurrence and severity of lameness and contact dermatitis in the control and BSFL broilers was moderately lower compared to previous large-scale studies on broiler leg health^50,51^, and one possible explanation for this could be the stocking density applied in our study. While stocking densities of 33 kg/m^2^ are applied commercially, it is the lower margin, and stocking densities up to 42 kg/m^2^ are allowed in the EU (Council Directive 2007/43/EC). Stocking densities below 35 kg/m^2^ have been linked to reduced incidence of foot pad dermatitis and hock burn^52^, and walking ability can be limited by higher stocking densities^53^. This is in line with the even lower incidence of these health issues in our previous study that applied a lower stocking density (14 kg/m^2^)^26^. However, in that study live BSFL provisioning did improve leg health. The absence of a treatment effect on leg health in the current study could be due to the higher stocking density combined with the relatively small pen size used. It is probable that, even though broilers performed more active behaviours, the large walking distances previously shown to improve broiler leg health (e.g. refs^41,48^) cannot be achieved in these conditions. This is in line with several previous studies that also applied small pens, where enrichment increased activity temporarily without affecting leg health^17,54^. In commercial-sized pens the activity increase caused by live BSFL provisioning could promote longer walking distances, potentially improving leg health. However, at higher commercial stocking densities the potential benefits of having larger pens might be negated by the limited space. Additional research in large-scale broiler housing systems is necessary to determine if and how live BSFL provisioning can benefit broiler welfare under these conditions.

The prevalence of tibial dyschondroplasia (TD) and white striping was higher compared to other studies^31,55,56^. For TD, the results are in line with a previous study that found a numerically higher occurrence in birds housed at 10 birds/m^2^, a similar stocking density as in the current study, compared to densities of 15 or 20 birds/m^2^, though the cause for this was unclear^57^. The relatively high occurrence of white striping could be a result of including only male broilers in the study, as white striping tends to be more common in male than female broilers^58^. The lack of effect of live BSFL provisioning on these health parameters could be due to the large influence of growth rate and weight on them^58,59^. The final weight was similar for broilers from all treatments, and it seems that the effect of growth could not be compensated by the increased activity caused by live larvae provisioning.

A strong risk factor for contact dermatitis is poor litter quality^4,7^, therefore the comparable moisture levels in the pens of all treatments could explain the absence of differences in foot pad dermatitis and hock burn. However, we expected that the increase in activity caused by live BSFL provisioning would improve the litter quality. Especially scratching behaviour, which was temporarily increased in broilers receiving 10% of their diet as live BSFL four times a day, can have this effect^60^, yet litter friability was lowest for this treatment. It is possible that the consumption of whole BSFL negatively affected litter quality. Whole BSFL contain high levels of fat, and animal fat cannot be digested well by young poultry^61^, meaning it might be excreted on the litter. This in turn can reduce the water uptake capabilities of the litter^62^. The negative effects of BSFL consumption might have outweighed the putative beneficial effects of activity on litter quality. If this is the case, then providing a lower amount of BSFL could be more beneficial for broiler litter quality, and in turn, broiler health.

## Conclusion

Our results indicate that live BSFL provisioning can reduce fearfulness and amplify broiler activity, especially foraging behaviour, and that this effect is most prominent in broilers with frequent or prolonged access to live BSFL. Feed intake decreased proportionally to the amount of BSFL, yet the final weight of broilers was not influenced by live BSFL provisioning. No positive or negative effects on broiler health were observed. While a reduction in fearfulness and facilitation of natural behaviour can already improve broiler welfare, live BSFL provisioning does have the potential to further benefit welfare by reducing health problems under intense commercial conditions in which health problems are more common.

## Supplementary information

Supplementary Information.

Supplementary Video.
