# Supplementary material for: Long-term access to live black soldier fly larvae (Hermetia illucens) stimulates activity and reduces fearfulness of broilers, without affecting health
Source: Sci Rep. 2020 Oct 15;10:17428. doi: 10.1038/s41598-020-74514-x (PMC7566458; doi:10.1038/s41598-020-74514-x)
Supplement: Supplementary file 1 — Supplementary Information. [file 41598_2020_74514_MOESM1_ESM.docx]

Long-term access to live black soldier fly larvae *(Hermetia illucens)* stimulates activity and reduces fearfulness of broilers, without affecting health

Allyson F. Ipema^1^*, Eddie A.M. Bokkers^2^, Walter J.J. Gerrits^3^, Bas Kemp^1^ & J. Elizabeth Bolhuis^1^

^1^ Adaptation Physiology Group, Department of Animal Sciences, Wageningen University & Research, P.O. Box 338, 6700 AH Wageningen, The Netherlands

^2^ Animal Production Systems Group, Department of Animal Sciences, Wageningen University & Research, P.O. Box 338, 6700 AH Wageningen, The Netherlands

^3^ Animal Nutrition Group, Department of Animal Sciences, Wageningen University & Research, P.O. Box 338, 6700 AH Wageningen, The Netherlands

*Correspondence and requests for materials should be addressed to A.I. (email: allyson.ipema@wur.nl).

**Supplementary Tables**

**Table S1.** Tube details.

| **Week** | **Tube diameter (mm)** | **Tube length (mm)** | **Hole diameter (mm)** | **Number of holes** |
| --- | --- | --- | --- | --- |
| 1 | 40 | 100 | 9 | 2 |
| 2 | 40 | 150 | 9 | 3 |
| 3 | 75 | 100 | 9 | 6 |
| 4-6 | 75 | 150 | 9 | 8 |

**Table S2.** Amounts and methods of live black soldier fly larvae (BSFL) provisioning in different treatments.

| **Treatment** | **% inclusion of BSFL in complete ration on dry matter basis** | **BSFL provisioning method per pen** | **Provisioning time (number of provisioning moments)** |
| --- | --- | --- | --- |
| Control | 0 | - | - |
| A5-S4 | 5 | Scattering | 08:00, 11:00, 14:00, 17:00 (4) |
| A5-S7 | 5 | Scattering | 08:00, 09:30, 11:00, 12:30, 13:00, 14:30, 17:00 (7) |
| A10-S4 | 10 | Scattering | 08:00, 11:00, 14:00, 17:00 (4) |
| A10-TB | 10 | Five tubes with holes | 08:00 (1, continuous access to tubes) |

**Table S3.** Visual representation of treatment and block distribution across experimental rooms.

| **Room 1** | | |  | **Room 2** | | |
| --- | --- | --- | --- | --- | --- | --- |
| Pen 1: Control |  | Pen 11: A5-S4 |  | Pen 21: A5-S4 |  | Pen 31: A10-S4 |
| Pen 2: A5-S7 |  | Pen 12: A10-S4 |  | Pen 22: A10-S4 |  | Pen 32: A5-S7 |
| Pen 3: A10-S4 |  | Pen 13: A5-S7 |  | Pen 23: A5-S7 |  | Pen 33: Control |
| Pen 4: A10-TB |  | Pen 14: A10-TB |  | Pen 24: A10-TB |  | Pen 34: A5-S4 |
| Pen 5: A5-S4 |  | Pen 15: Control |  | Pen 25: Control |  | Pen 35: A10-TB |
| Pen 6: A5-S7 |  | Pen 16: A5-S4 |  | Pen 26: A10-S4 |  | Pen 36: A10-S4 |
| Pen 7: Control |  | Pen 17: A10-S4 |  | Pen 27: A5-S7 |  | Pen 37: A5-S4 |
| Pen 8: A5-S4 |  | Pen 18: A10-TB |  | Pen 28: A5-S4 |  | Pen 38: A5-S7 |
| Pen 9: A10-TB |  | Pen 19: Control |  | Pen 29: Control |  | Pen 39: A10-TB |
| Pen 10: A10-S4 |  | Pen 20: A5-S7 |  | Pen 30: A10-TB |  | Pen 40: Control |

Treatments include broilers receiving no larvae (Control), or provided with live larvae in different amounts (5 or 10 % of the total dietary DM replaced with larvae, A5 and A10 respectively) and provisioning methods (scattered four or seven times a day, S4 and S7 respectively, or in tubes, called TB). Blocks are bordered by solid lines. Each block contains five pens with five different treatments in a randomized order.

**Table S4 A.** Ingredients and (analysed) chemical composition of dietary components.

|  | **Starter feed^1,5^** | **Core feed^2, 3, 5^** | **BSFL-replacer mix^2,4,5^** | **BSFL^2,6^** |
| --- | --- | --- | --- | --- |
| *Ingredients (g/kg)* | | | | |
| Corn | 368.1 | 225.1 | - | - |
| Wheat | 250.0 | 409.6 | - | - |
| Soybean meal | 290.0 | 201.3 | - | - |
| Fishmeal | - | 69.9 | 160 | - |
| Rapeseed meal | 40.0 | 33.2 | - | - |
| Potato protein | - | - | 350 | - |
| Sunflower oil | 16 | 33.6 | - | - |
| BSFL oil |  | - | 350 | - |
| Premix | 5 | 5.6 | - | - |
| Lime fine | 14 | - | - | - |
| Monocalcium phosphate | 8.3 | 4.5 | 11.0 | - |
| Salt | 2.1 | 2.3 | - | - |
| CaCO3 | - | 10.6 | - | - |
| NaHCO_3_ | 1.8 | 1.2 | - | - |
| Phytase | 0.2 | 0.2 | - | - |
| DL-methionine | 2.1 | 2.1 | - | - |
| L-threonine | 0.45 | 0.6 | - | - |
| L-valine | 0.1 | 0.1 | - | - |
| L-lysine HCl | 1.85 | - | - | - |
| Diamol | - | - | 129 | - |
| *Chemical composition* | | | | |
| Dry matter (DM, g/kg) | 876.8 | 883 | 900 | 350.7 |
| ME (MJ/kg of DM) | 12.46 | 11.53 | 19.5 | 19.5 |
| Crude protein (g/kg of DM) | 220 | 223 | 419 | 419 |
| Crude fat (g/kg of DM) | 36.5 | 80 | 371 | 371 |
| Calcium (g/kg of DM) | 9 | 7.6 | 6.3 | 6.3 |
| Phosphorus (g/kg of DM) | 7.1 | 6.0 | 6.8 | 6.8 |

^1^Provided to all broilers during the starter period (day 1-7). ^2^Provided in different combinations during the grower period (day 8-42). ^3^Core diet component of the grower feed supplied to all broilers. ^4^Diet component with similar chemical composition as BSFL. ^5^Chemical composition according to CVB (2006). ^6^Chemical composition based on analysis via standardized protocols (DM 10032, Protein 10005, Fat 10112, Calcium and Phosphorus 10040).

**Table S4 B.** (Chemical) composition of grower feed. Control, A5 and A10: 0 %, 5 % and 10 %, respectively, of the total dietary dry matter replaced with live larvae. Distributions of diet components are based on larval supply, to provide all broilers with iso-energetic (based on ME) diets with a similar chemical composition.

|  | **Control** | **A5** | **A10** |
| --- | --- | --- | --- |
| *Dietary component, % of total DM intake* | | | |
| Core feed | 90 | 90 | 90 |
| BSFL-replacer mix | 10 | 5 | 0 |
| BSFL | 0 | 5 | 10 |
| *Chemical composition* | | | |
| ME (MJ/kg of DM) | 12.4 | 12.3 | 12.3 |
| Crude protein (g/kg of DM) | 244 | 243 | 243 |
| Crude fat (g/kg of DM) | 111 | 111 | 110 |
| Calcium (g/kg of DM) | 7.5 | 7.5 | 7.5 |
| Phosphorus (g/kg of DM) | 6.0 | 6.0 | 6.1 |

**Table S5.** Health scores protocol.

| **Measure** | **Score** | **Description** | **Reference** |
| --- | --- | --- | --- |
| Gait | 0 | Normal, dextrous and agile. | (Butterworth, 2009) |
|  | 1 | Slight abnormality, but difficult to define. |  |
|  | 2 | Definite and identifiable abnormality. |  |
|  | 3 | Obvious abnormality, affects ability to move. |  |
|  | 4 | Severe abnormality, only takes a few steps. |  |
|  | 5 | Incapable of walking. |  |
| Hock burn^1^ | 0 | No evidence of hock burn. | (Butterworth, 2009) |
|  | 1 | Minimal evidence of hock burn. |  |
|  | 2 | Minimal evidence of hock burn. |  |
|  | 3 | Evidence of hock burn. |  |
|  | 4 | Evidence of hock burn. |  |
| Food pad dermatitis^1^ | 0 | No lesions. | (Butterworth, 2009) |
|  | 1 | Raised central pad, reticulate scales are separated, with or without small, black necrotic area(s). |  |
|  | 2 | Marked swelling of the foot pad, black reticulate scales forming scale-shaped necrotic areas, with necrosis evident on less than one-quarter of the total foot pad area. |  |
|  | 3 | Marked swelling and enlargement of the entire foot pad, necrosis extending up to one-half of the total foot pad area. |  |
|  | 4 | Marked swelling and enlargement of the entire foot pad, necrotic cells covering more than one-half of the total foot pad area. |  |
| Cleanliness^1^ | 0 | The feathers and skin are completely clean. | (Butterworth, 2009) |
|  | 1 | The feathers and skin on the belly of the bird are slightly dirty. |  |
|  | 2 | The feathers and skin on the belly of the bird are quite dirty. Dirt is caked on the feathers. Other parts of the plumage may be slightly dirty. |  |
|  | 3 | The feathers and skin on the belly of the bird are quite dirty. Dirt is caked on the feathers and also the rest of the plumage (back, wings is visibly dirty). |  |
| Thigh scratches | Absent | No thigh scratches present on both thighs. |  |
|  | Present | One or more thigh scratches present on one or both thighs. |  |
| Wooden breast | Absent | Soft breast muscle. |  |
|  | Mild | Part of the breast muscle is hardened. |  |
|  | Severe | Almost the whole area of the breast muscle is hardened. |  |
| White striping^1^ | 0 | No distinct white lines. | (Kuttappan, Hargis, & Owens, 2016) |
|  | 1 | Small white lines, generally <1 mm thick, but apparently visible on the filet surface. |  |
|  | 2 | Large white lines (1-2 mm thick) very visible on the filet surface. |  |
|  | 3 | Thick white bands (>2 mm thickness) covering almost entire filet surface. |  |
| Abdominal fluid | Absent | No abdominal fluid present. |  |
|  | Present | Abdominal fluid present. |  |
| Tibial dys-chondroplasia^1^ | 0 | No lesion. | (Karaarslan & Nazlıgül, 2018) |
|  | 1 | Lesion less than 0.5 cm in size. |  |
|  | 2 | Lesion between 0.5-1 cm in size. |  |
|  | 3 | Lesion more than 1 cm in size. |  |

^1^See reference for detailed illustrations of individual scores.
